# Neurodegeneration in *Drop-Dead* Mutant *Drosophila melanogaster* Is Associated with the Respiratory System but Not with Hypoxia

**DOI:** 10.1371/journal.pone.0068032

**Published:** 2013-07-10

**Authors:** Christine Lynn Sansone, Edward M. Blumenthal

**Affiliations:** Department of Biological Sciences, Marquette University, Milwaukee, Wisconsin, United States of America; University of California Los Angeles, United States of America

## Abstract

Mutations in the gene *drop-dead* (*drd*) cause diverse phenotypes in adult *Drosophila melanogaster* including early lethality, neurodegeneration, tracheal defects, gut dysfunction, reduced body mass, and female sterility. Despite the identification of the *drd* gene itself, the causes of early lethality and neurodegeneration in the mutant flies remain unknown. To determine the pattern of *drd* expression associated with the neurodegenerative phenotype, knockdown of *drd* with various Gal4 drivers was performed. Early adult lethality and neurodegeneration were observed upon knockdown of *drd* in the tracheal system with two independent insertions of the *breathless-Gal4* driver and upon knockdown in the tracheal system and elsewhere with the *DJ717-Gal4* driver. Surprisingly, rescue of *drd* expression exclusively in the tracheae in otherwise mutant flies rescued the neurodegenerative phenotype but not adult lethality. Gut dysfunction, as measured by defecation rate, was not rescued in these flies, and gut function appeared normal upon tracheal-specific knockdown of *drd*. Finally, the hypothesis that tracheal dysfunction in *drd* mutants results in hypoxia was tested. Hypoxia-sensitive reporter transgenes (*LDH-Gal4* and *LDH-LacZ*) were placed on a *drd* mutant background, but enhanced expression of these reporters was not observed. In addition, manipulation of *drd* expression in the tracheae did not affect expression of the hypoxia-induced genes *LDH*, *tango*, and *similar*. Overall, these results indicate that there are at least two causes of adult lethality in *drd* mutants, that gut dysfunction and neurodegeneration are independent phenotypes, and that neurodegeneration is associated with tracheal expression of *drd* but not with hypoxia.

## Introduction

While neurodegenerative diseases impact the lives of millions, the underlying mechanisms of many such diseases remain poorly understood. *Drosophila melanogaster* has emerged as a powerful model organism for the study of neurodegeneration. The first neurodegenerative mutant discovered in *Drosophila* was in the gene *drop-dead* (*drd*) [1], so named due to the short lifespan phenotype these mutants exhibit. Flies with the strong alleles *drd^lwf^* or *drd^1^* have a median survival of 4 days and all flies die within 3 weeks of eclosion [1]–[3]. Despite the early discovery of *drd* mutants, the function of the *drd* gene and the cause of neurodegeneration remain unknown.

Two hypotheses have been advanced to explain neurodegeneration in *drd* mutants. At the time of eclosion, the brains of mutant flies appear normal except that the glial cells remain morphologically immature. Within a few days, the flies begin to display locomotor defects and at this point there is gross degeneration of the brain. It is hypothesized that the immature glial cells are responsible for the subsequent neurodegeneration [3]. However, it remains unknown how mutations in *drd* cause immature glial cells or how immature glial cells might cause neurodegeneration. The other hypothesis for the cause of neurodegeneration involves hypoxia. In *drd* mutants, the respiratory tracheal system is fragile and eventually collapses [4]–[6]. This collapse is observed prior to neurodegeneration [6]. A collapsed tracheal system is predicted to cause hypoxia, which would lead to neurodegeneration [4]–[6]. Consistent with this hypothesis, hypoxia-responsive genes were shown by semi-quantitative RT-PCR to be upregulated in 5 day old *drd^1^* mutants [4]. It should be noted that these two hypotheses are not mutually exclusive. For example, it is possible that collapse of the pupal tracheal system could cause hypoxia, and that this hypoxic insult would result in glial cells failing to mature and subsequent neurodegeneration.

In addition to neurodegeneration and short lifespan, *drd* mutants display several other phenotypes that appear to be unrelated. The mutants display a gut phenotype, in which food remains in the crop and it is unable to move into the midgut for digestion to occur. This causes a depletion of triglyceride and glycogen stores, indicating that the flies may be starving [7]. Additionally, *drd* mutants have a reduced body size and homozygous females are sterile [1], [2]. We have begun the process of separating these phenotypes to understand the causal relationships among them. By knocking down and rescuing *drd* expression at specific developmental stages, we have separated the shortened lifespan and reduced body mass phenotypes. For survival, *drd* expression is both necessary and sufficient during mid to late metamorphosis, indicating that *drd* is an essential developmental gene. In contrast, the attainment of normal adult body mass requires *drd* expression during a broader period of development [8].

The protein product of *drd* is a member of the NRF (nose resistant to fluoxetine) family of proteins. Proteins in this family contain a cysteine-rich NRF domain and have limited homology to a family of bacterial acyltransferases [2], [9]. Drd is localized to a membrane bound organelle compartment and predicted to be an integral membrane protein [2], [4]. There is no reported biochemical function of Drd or the other 16 NRF proteins in the *Drosophila* genome.

The goal of this study is to determine the relationship between neurodegeneration and hypoxia in *drd* mutants. Utilizing tissue-specific knockdown and rescue, we determined that *drd* expression is necessary but not sufficient in the tracheae to prevent the early lethality phenotype. However, expression in the tracheal system is both necessary and sufficient to prevent neurodegeneration. Surprisingly, hypoxia-sensitive reporter transgenes indicated that *drd* mutant flies are not detectably hypoxic. In light of these results, a new hypothesis for the cause of neurodegeneration in *drd* mutants is proposed.

## Materials and Methods

### *Drosophila* Stocks and Maintenance

All fly stocks were maintained on standard cornmeal-yeast-agar food (http://flystocks.bio.indiana.edu/Fly_Work/media-recipes/molassesfood.htm) at 25°C on a 12 h:12 h light:dark cycle. RNAi experiments used the 51184 UAS-Dcr-2 and UAS-Dcr-2 37404 lines created previously by recombination between VDRC stocks *w^1118^;P{GD3367}v37404* (FBst0461992) and *w^1118^; P{GD15915}v51184* (FBst0469325) and Bloomington stock *w^1118^; P{UAS-Dcr-2.D}2* (FBst0024650) [8]. Other stocks (*w^1118^; P{GAL4}repo/TM3, Sb^1^* (FBst0007415), w^*^; *P{GawB}17A/CyO* (FBst0008474), w^*^; *P{GAL4-btl.S}2, P{UASp-Act5C.T:GFP}2/CyO, P{lacZ-un8}276* (FBst0008807, referred to as *btl-Gal4(II)*), y^1^ w^*^; P{Act5CGAL4}17bFO1/TM6B, Tb^1^ (FBst0003954), w^*^; P{UAS-lacZ.B}Bg4-2-4b(FBst0001777), *w^1118^; P{GawB}DJ717* (FBst0008180), and y^1^ w^*^; wg^Sp-1^/CyO; P{btl-moe.mRFP1}3, P{GAL4-btl.S}3-1, P{UAS-mCD8::GFP.L}LL6/TM6B, Tb^1^ (FBst0041803, referred to as *btl-Gal4(III)*)) were obtained from the Bloomington Drosophila Stock Center. The *w; elav-Gal4* and *w; UAS-GFP* lines were gifts from Jay Hirsh. The *LDH-LacZ* and *LDH-Gal4 UAS-GFP* lines were gifts from Pablo Wappner and Jon Harrison. The line bearing the *UAS-drd* on the second chromosome on a *drd^lwf^* background was described previously [8]. The genes and alleles referenced in this work include *drd* (FBgn0260006), *drd^lwf^* (FBal0193421), *similar* (FBgn0015542), *tango* (FBgn0264075), *lactate dehydrogenase* (FBgn0001258), and *rp49* (FBgn0002626). Stocks were not outcrossed prior to this study.

For rescue experiments with two copies of the *btl-Gal4(II)* driver, the *UAS-drd* transgene was recombined onto the *btl-Gal4(II)* driver chromosome by a standard crossing scheme. Recombinants were identified by PCR (GoTaq Hot Start Polymerase, Promega, Madison, WI). Primers used to detect the *UAS-drd* transgene were pUAST 3′ seq: 5′ CAG TTC CAT AGG TTG GAA TC 3′ and CG5652 6a: 5′ GAT CGC CTG GTG TTT GTT TT 3′, and primers used to detect the *btl-Gal4* transgene were Gal4 F: 5′ GGC TAG AAA GAC TGG AAC AGC T 3′, and Gal4 R: 5′ AGG GCA AGC CAT CCG ACA TG 3′. The resulting recombinant chromosome was crossed onto a *drd^lwf^* background.

### Lifespan Assays

Flies were collected on the day of eclosion, transferred to fresh vials every 2–7 days, and scored daily for survival for 40 days. A minimum of 50 flies per genotype were used for each survival curve.

### Anoxic Treatment of *Drosophila*

Vials of flies were placed into a BD GasPak EZ Anaerobe Gas Generating Pouch System with Indicator (Becton, Dickinson and Company, Sparks, MD) for 5 hours, resulting in an anoxic exposure (≤1% O_2_). Assays were performed immediately upon removal of flies from the pouch.

### Synthesis of cDNA and Quantitative Real-Time PCR

RNA was isolated from whole flies using Trizol reagent (Life Technologies, Grand Island, NY). One µg total RNA was treated with RQ1 RNase-free DNase (Promega) for 30 minutes at 37°C and then cDNA synthesis was performed with qScript cDNA supermix (Quanta Biosciences, Gaithersburg, MD).

Real-time PCR was performed on cDNA using a MyiQ thermocycler (Bio-Rad, Hercules, CA) and PerfeCta SYBR Green FastMix for iQ (Quanta Biosciences). Each sample was run in triplicate. A melt curve was performed directly after amplification to verify the authenticity of the PCR products. Experimental transcript levels (*sima*, *tango*, or *LDH*), relative to the housekeeping gene *rp49*, were calculated in MyiQ software (Bio-Rad) using a dilution series of whole fly cDNA that was included in each PCR run. Primers used: *rp49* F: 5′ AAG ATC GTG AAG AAG CGC ACC AA 3′, *rp49* R: 5′ CTG TTG TCG ATA CCC TTG GGC TT 3′, *Sima* F: 5′ AGC CCA ATC TGC CGC CAA CC 3′, *Sima* R: 5′ TGG AGG CCA GGT GGT GGG AC 3′, *Tango* F: 5′ CGG CTG CTC ATA CGC CCG AG 3′, *Tango* R: 5′ GCC CAG CAT GTG CGT CTG GT 3′, *LDH* F: 5′ CTA CAC GAT CCA TTC GCA ACA CC 3′, and *LDH* R: 5′ ACT TGA TGC TAC GAT TCG TGG 3′.

### Defecation Assays

Assays were performed as previously described [2]. Briefly, two male flies were placed in a vial containing instant food (Carolina Biological, Burlington, NC) prepared with 0.5% Acid Blue 9 on the day of eclosion. After 24 hours, the flies were transferred to a fresh vial. After another 24 hours, the blue fecal spots on the vial were counted.

### Protein, Triglyceride, and Glycogen Assays

Homogenates from P0–P4 male flies were prepared and assayed for protein, triglyceride, and glycogen concentration levels as previously described [7]. Triglyceride and glycogen levels were normalized to protein concentration. For each assay, homogenates were analyzed in triplicate and concentration standards in duplicate. All spectrophotometric assays were carried out with a Multiskan Ascent plate reader (ThermoFisher Scientific, Waltham, MA) and analyzed with Ascent v2.6 software (ThermoFisher).

### Haematoxylin and Eosin Staining

Flies were decapitated and heads were fixed in 4% paraformaldehyde in 1x PBS for 3 hours at 4°C. Heads were washed 3 times for 10 minutes in 1x PBS and then were incubated in 30% sucrose in 1x PBS at 4°C overnight. Heads were mounted in Tissue-Tek OCT Compound (Sakura, Toyko, Japan), snap frozen, and sectioned at 5 µm. Sections were stained with haematoxylin (VWR, West Chester, PA) and eosin (VWR), mounted in Permount (Electron Microscopy Sciences, Hatfield, PA), and imaged on Axioskop-2 (Zeiss, Thornwood, NY) at 10x with Axiovision image analysis software (Zeiss). Neurodegeneration was scored by the presence of holes in intact brain tissue. Multiple sections of a single brain were viewed and the presence or absence of holes was consistent throughout the whole tissue.

### Visualization of GFP Reporter Expression

Legs were removed and mounted in VectaShield Mounting Medium with DAPI (Vector Laboratories, Burlingame, CA). Samples were immediately imaged on a Nikon A1 Confocal Microscope (Nikon, Tokyo, Japan) at 10x with NIS-Elements AR software (Nikon).

### Visualization of Pupal Tracheae

4-day-old pupae were dissected out of their pupal cases. Pupae were placed in 90% glycerol/0.3x PBS for 30 min. Samples were imaged on a Nikon A1 Confocal Microscope at 10x with NIS-Elements AR software.

### β-galactosidase Assays

β-galactosidase assays were performed as previously described [10]. Three flies were homogenized in 100 µL of assay buffer (50 mM KH_2_PO_4_, 1 mM MgCl_2_, pH 7.5) and taken to a final volume of 1 mL. Samples were briefly vortexed and centrifuged at 2500 rpm for 2 minutes at 4°C. Supernatant was recovered and the protein concentration determined. Enzymatic reactions were performed by incubating 100 µg of protein with 1 mM chlorophenol red-β-D-galactopyranoside (Sigma-Aldrich) at 37°C in the dark. The optical density was read continuously at 574 nm and recorded every 5 seconds on a Shimadzu UV-1800 spectrophotometer (Kyoto, Japan).

### Statistics and Data Analysis

Data were graphed and analyzed using GraphPad Prism v5 for Windows (GraphPad Software, San Diego, CA, www.graphpad.com). For survival curves, pair-wise comparisons of each experimental group with its sibling control were carried out using a Mantel-Haenszel test. For triglyceride and glycogen assays, a 2-way ANOVA was performed. For defecation assays and qRT-PCR, a 1-way ANOVA and Bonferroni’s post-hoc test were performed between each experimental group and its appropriate control. For β-galactosidase assays, data were graphed using KaleidaGraph 4.1 (Synergy Software, Reading, PA, www.synergy.com).

## Results

**Figure 1:**
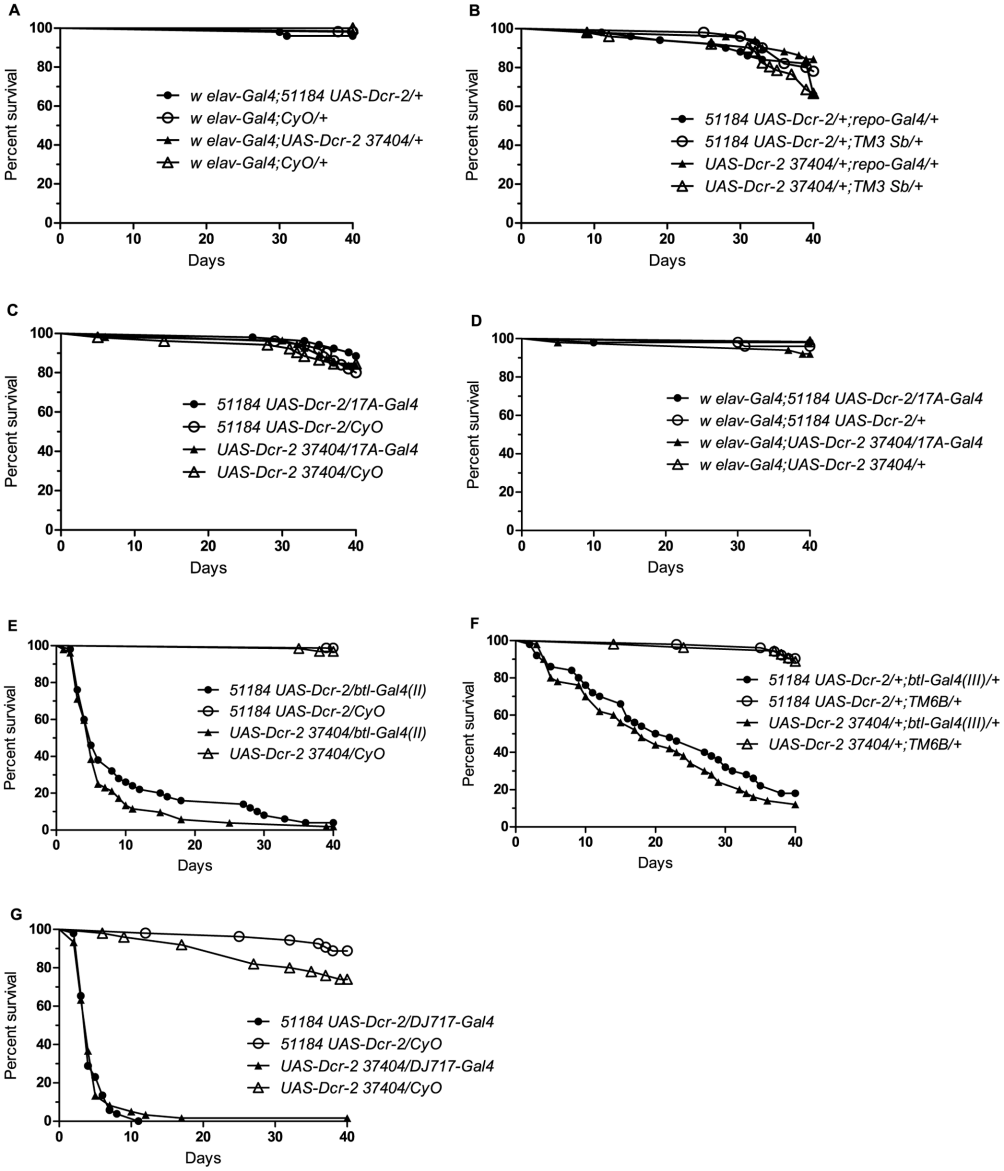
***drd*** **expression is required in the tracheae but not the brain for survival.** There was no effect on lifespan when *drd* expression was knocked down by either *w;{GD15915}v51184 UAS-Dcr-2 *or *w;UAS-Dcr-2 {GD3367}v37404* with the *elav-Gal4* (a), *repo-Gal4* (b), *17A-Gal4* (c), or *elav-Gal4; 17A-Gal4* (d) drivers. Early lethality was observed when *drd* expression was knocked down with the *btl-Gal4(II)* (e), *btl-Gal4(III)* (f), and *DJ717-Gal4* (g) drivers. n = 50–81 flies/genotype.

We have previously demonstrated that targeted knockdown and rescue of *drd* expression can recapitulate and rescue, respectively, early adult lethality [8]. We utilized this system to determine the tissues necessary for the neurodegenerative phenotype observed in *drd* mutants. We assumed that any manipulation of *drd* expression that causes neurodegeneration would result in early lethality. Therefore, we crossed various Gal4 drivers with the two UAS-Dcr2-RNAi transgenes directed against *drd* (51184 and 37404) and scored for survival. Knocking down *drd* expression with the neuronal driver *elav-Gal4* failed to cause early lethality (measured in the first 40 days after eclosion) with either RNAi line for the duration of the experiment (Fig. 1a). Sibling controls that lacked the UAS-Dcr-2-RNAi transgene also survived the duration of the experiment (p = 0.50 for 51184 and p = 0.29 for 37404 for pairwise comparisons of each knockdown population with its sibling control). Additionally, knockdown of *drd* in glial cells with the *repo-* and *17A-Gal4* drivers did not have an effect on lifespan (Fig. 1b, *repo* p = 0.26 for 51184 and p = 0.60 for 37404; Fig. 1c, *17A* p = 0.24 for 51184 and p = 0.94 for 37404). It was previously hypothesized that *drd* expression might be required in both glia and neurons to allow signaling between these two cell types [3]. Therefore, we knocked down the expression of *drd* in both neurons and glia together (*elav-Gal4; 17A-Gal4*), and again, there was no significant effect on survival (Fig. 1d, p = 0.5445 for 51184 and p = 0.1055 for 37404). However, knockdown of *drd* with the tracheal specific driver, *breathless* (*btl-Gal4(II)*), resulted in a significant difference in lifespan (Fig. 1e). Both UAS-RNAi lines displayed a median survival of 5 days, while sibling controls that lacked the Gal4 driver survived the duration of the experiment (p<0.0001 for both RNAi lines). To visualize the expression pattern of the *btl-Gal4(II)* driver, we took advantage of a UAS-GFP transgene present on the same chromosome as the driver. The observed pattern of fluorescence on the final day of metamorphosis was consistent with tracheal-specific expression of *btl-Gal4(II)*, as previously reported [11], [12] (Fig. S1a). To control for possible position effects, we also tested a *btl-Gal4* driver inserted on the third chromosome (*btl-Gal4(III)*). Knockdown of *drd* expression with this driver caused a significant difference in lifespan, with a median survival of 21 days for 51184 and 18 days for 37404 (Fig. 1f, p<0.001 for both RNAi lines). Knocking down expression of *drd* with another driver, *DJ717-Gal4* [13], also caused early lethality (Fig. 1g). Both UAS-RNAi lines displayed a median survival of 4 days (p<0.0001 for both RNAi lines). Consistent with the *btl-Gal4(II)* expression pattern results, we also observed expression in the tracheae, among other tissues, in late pupae with the *DJ717* driver (Fig. S1c). From these experiments we conclude that *drd* expression is required in the tracheal system for adult survival.

**Figure 2:**
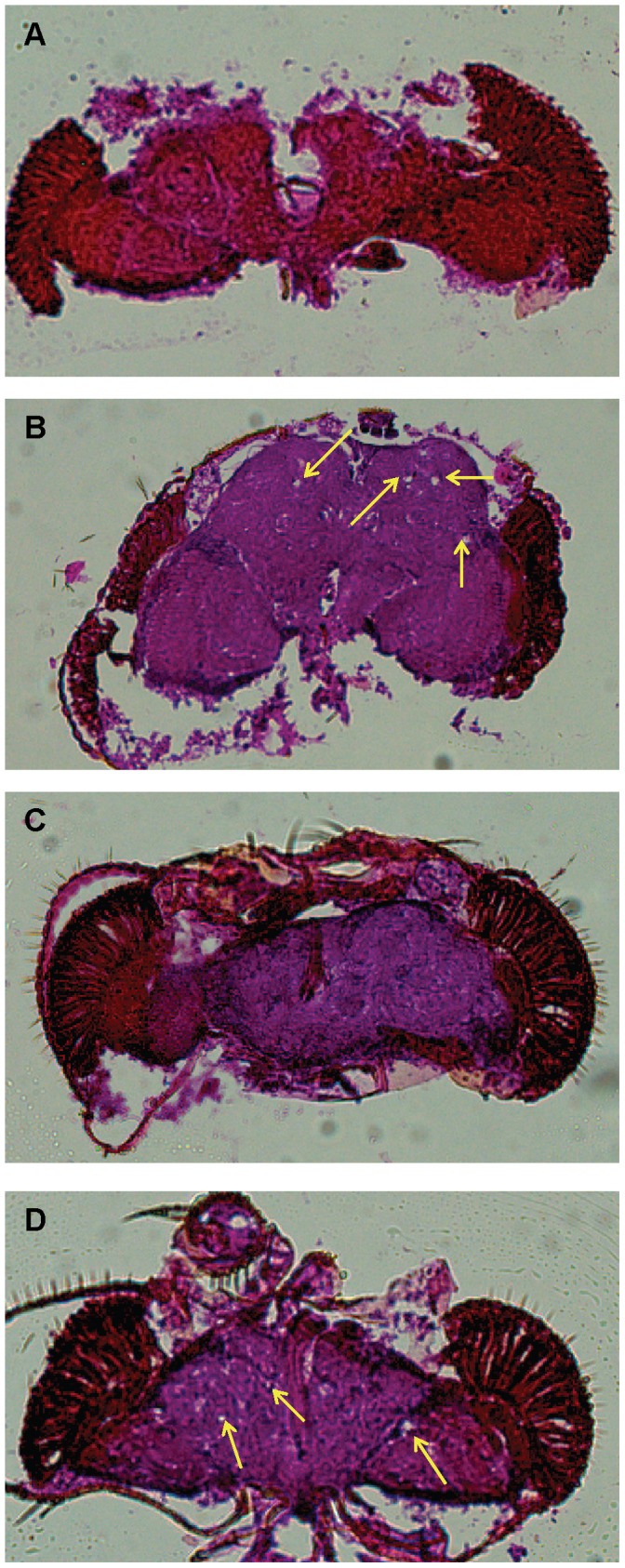
Knockdown of ***drd*** **in the tracheae causes neurodegeneration.** Brain sections of 4 day old flies were stained with haematoxylin and eosin. Neurodegeneration was observed in *btl-Gal4(II)/51184 UAS-Dcr-2* (b) and *btl-Gal4(II)/UAS-Dcr-2 37404* (d), but not in the sibling controls (a and c, respectively). Arrows indicate holes.

We wanted to determine if knockdown of *drd* in the tracheae is also responsible for neurodegeneration. Heads of flies at four days post-eclosion were sectioned and stained with haematoxylin and eosin. Since the *btl-Gal4(II)* driver displays a stronger knockdown phenotype than the *btl-Gal4(III)* driver, the second chromosome driver was used for all subsequent experiments. In the *btl-Gal4(II)* knockdown flies, holes were observed in 6 of 9 brains with the 51184 line and 5 of 9 brains with the 37404 line (Fig. 2b and 2d, respectively). However, no degeneration was observed in sibling controls (Fig. 2a, 0 of 7 brains and 2c, 0 of 11 brains). Additionally, neurodegeneration was observed in *DJ717-Gal4* knockdown flies. Holes were observed in 7 of 8 brains with the 51184 line and 6 of 9 brains with the 37404 line (Fig. S2b and S2d, respectively). Again, the brains of the sibling control flies were intact (Fig. S2a, 0 of 6 brains and S2c, 0 of 8 brains). Therefore, knockdown of *drd* in the tracheae results in neurodegeneration.

**Figure 3:**
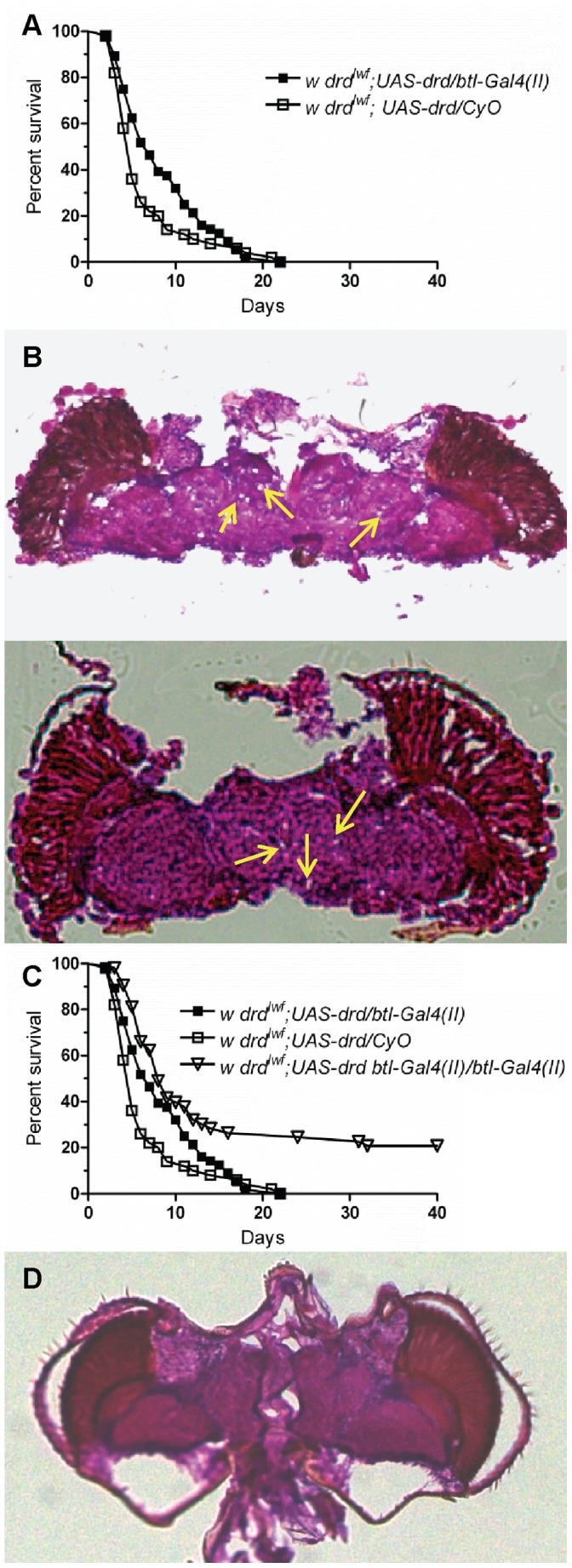
Expression of ***drd*** **in the tracheae does not rescue survival, but does rescue neurodegeneration.** Tracheal specific expression of *drd* by crossing *btl-Gal4(II)/CyO* males with *drd^lwf^/FM7a;UAS-drd* females did not rescue the early lethality phenotype (a) or the neurodegeneration phenotype (b: Control on top, degeneration was observed in 7 of 7 brains; Rescue on bottom, degeneration was observed in 7 of 7 brains). Two copies of the *btl-Gal4(II)* driver where utilized and the above experiments repeated. *drd^lwf^;UAS-drd btl-Gal4(II)/btl-Gal4(II)* flies still exhibited the early lethality phenotype. Single copy Gal4 rescue and sibling controls are the same data as in Fig. 2a on this graph (c). However, these flies no longer display neurodegeneration (d). n = 0 of 3 degenerating brains. Neurodegeneration was assayed as in Figure 2. For survival curves, n = 50–56 flies/genotype.

Because we identified tracheal expression of *drd* as necessary with regard to survival and neurodegeneration, we next tested whether expression in this tissue is sufficient. We utilized the *UAS-drd* rescue construct on a *drd^lwf^* background to drive tissue specific expression of *drd* in the tracheae. Expressing *drd* in the tracheae alone resulted in a survival curve that just failed to reach statistical significance compared to sibling controls (p = 0.06) but clearly did not rescue early adult lethality (Fig. 3a). Rescue flies survived for a median of 7 days and the sibling controls without the Gal4 driver survived for 5 days. Additionally, sectioning brains of 7 day old tracheal rescue flies still revealed neurodegeneration (Fig. 3b). To determine whether these results stemmed from an insufficient level of *drd* expression, we repeated the rescue with two copies of the Gal4 driver. This level of *drd* overexpression should not have deleterious effects, since we have previously reported that ubiquitous overexpression of *drd* (32-fold overexpression) resulted in no observable phenotypes [8]. Rescue flies with two copies of the *btl-Gal4(II)* driver had a median survival of 8 days (Fig. 3c). While this was a significant difference compared to both sibling controls without a Gal4 driver (p<0.001) and rescue flies with a single copy of the driver (p = 0.0015), the early lethality phenotype was not rescued in the large majority of flies. However, the addition of a second copy of the *btl-Gal4(II)* driver did fully rescue the neurodegenerative phenotype in 8 day old flies (Fig. 3d).

**Figure 4:**
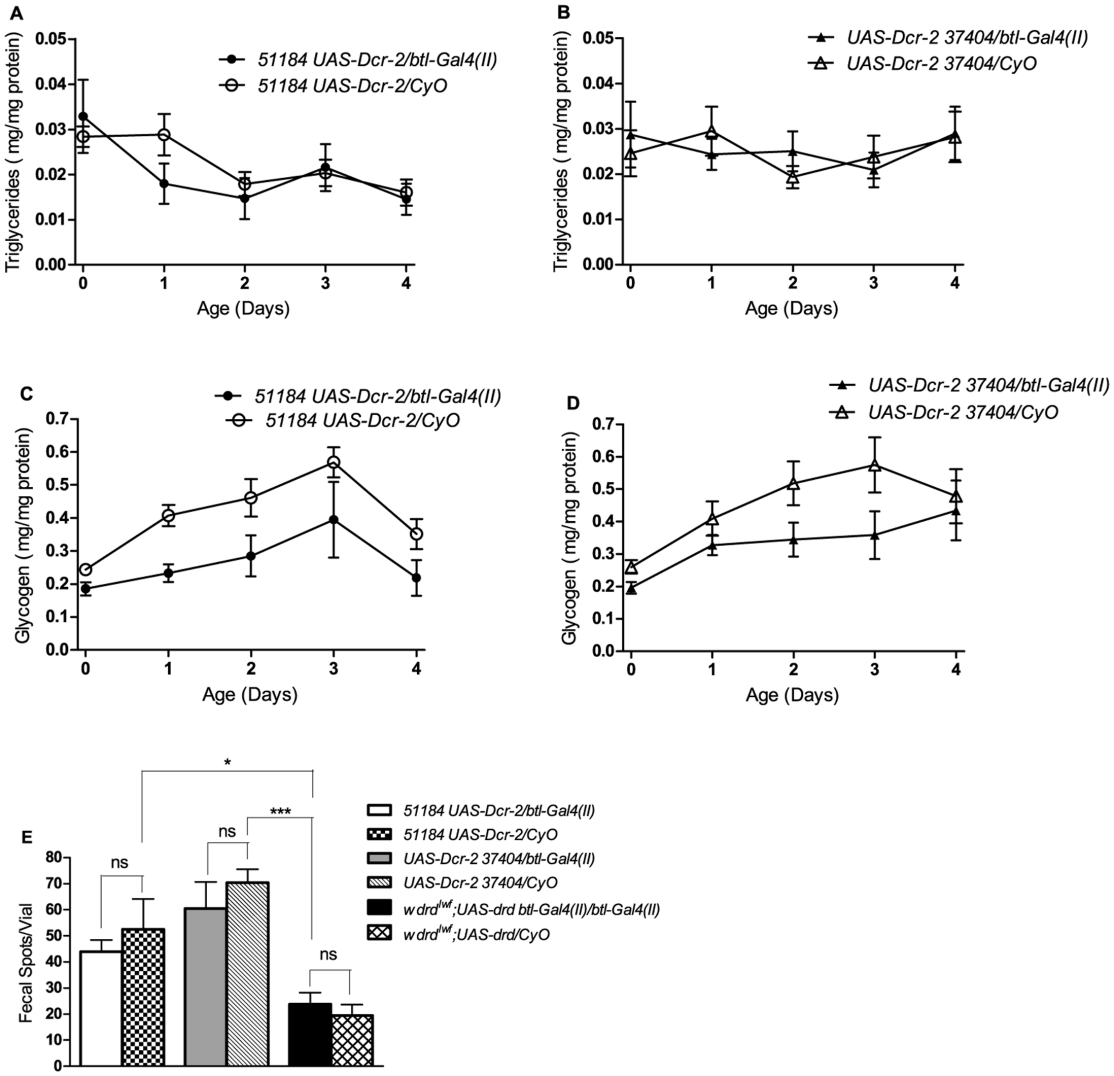
Knockdown of ***drd*** **in the tracheae does not cause starvation.** Triglyceride (a,b) and glycogen (c,d) levels of male progeny from crossing *btl-Gal4/CyO* females with either *w;{GD15915}v51184 UAS-Dcr-2* (a,c) or *w;UAS-Dcr-2 {GD3367}v37404* (b,d) males. No significant effect of genotype was seen by 2-way ANOVA. n = 10 flies/point. Pairs of male flies of the same genotype from the cross above were also utilized for defecation assays during the second day post-eclosion. Defecation assays were also performed on *drd^lwf^;UAS-drd btl-Gal4(II)/btl-Gal4(II)* (e). Asterices indicate significant difference by 1-way ANOVA and Bonferroni’s post-test. ns p>0.05; *p<0.05; ***p<0.001. n = 6 pairs of flies/condition. Error bars represent SEM.

Since rescue of *drd* expression in the *btl* pattern prevents neurodegeneration but not early lethality, we hypothesized that there is an additional cause of early lethality in *drd* mutants that is not associated with the respiratory system. To examine the role of the previously reported defect in gut function, we measured triglyceride and glycogen levels following tracheal knockdown of *drd* and in sibling controls during the first 5 days of adult life. Both triglyceride (Fig. 4a and b) and glycogen (Fig. 4c and d) stores were not significantly different between knockdown flies and sibling controls (two-way ANOVA, p>0.05). Additionally, these flies did not exhibit a significant difference in defecation, suggesting no defect in the movement of food through the gut (Fig. 4e). Therefore, flies with *drd* knocked down in the tracheal system do not exhibit the gut phenotype of *drd* mutants. We next examined *btl* rescue flies. Both experimental flies with two copies of the *btl-Gal4(II)* driver and sibling controls displayed a reduced production of fecal spots, suggesting that rescue of *drd* expression in the tracheae is not sufficient to rescue food movement through the digestive tract (Fig. 4e).

**Figure 5:**
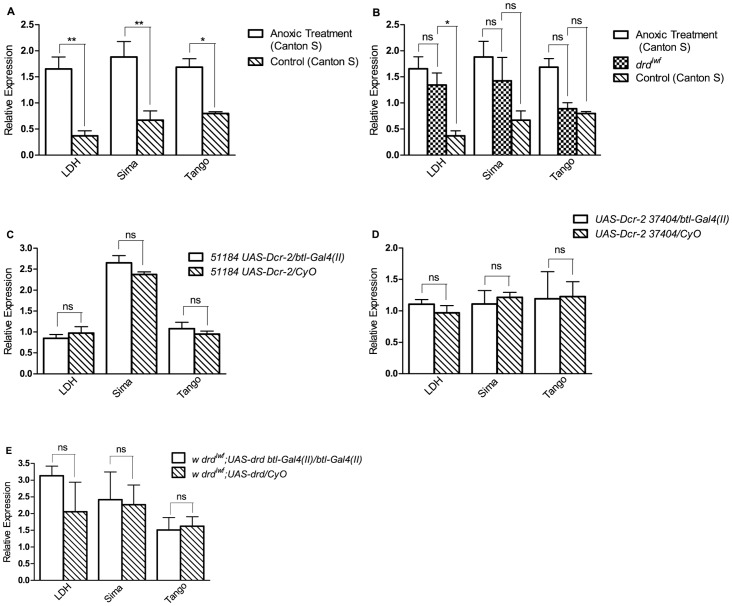
Hypoxia-induced genes are not upregulated in ***drd*** **mutants or affected by tracheal expression of** ***drd*** **.** *LDH*, *sima*, and *tango* are all upregulated after 5 hours of anoxic treatment in Canton S flies (a) while the genes are not consistently upregulated in *drd^lwf^* males (b). Control and anoxic treated data are the same in Fig. 5a and 5b. Male *51184 UAS-Dcr-2/btl-Gal4(II)* (c), *UAS-Dcr-2 37404/btl-Gal4(II)* (d), and *w drd^lwf^;UAS-drd btl-Gal4(II)/btl-Gal4(II)* flies (e) were assayed to determine the expression levels of hypoxia-induced genes. Asterices indicate significant difference by 1-way ANOVA and Bonferroni’s post-test between experimental and sibling controls in a,c,d,e and between control or anoxic Canton S and *drd^lwf^* in b. ns p>0.05; *p<0.05; **p<0.01. n = 3. Error bars represent SEM.

As it has previously been reported that *drd* mutants are hypoxic [4], we next investigated the link between tracheal expression of *drd* and hypoxia. Quantitative RT-PCR was performed to determine if hypoxia-induced genes are upregulated upon tracheal knockdown of *drd* and downregulated upon tracheal rescue of *drd*. *Lactate dehydrogenase* (*LDH*), *similar* (*sima*), and *tango* have all previously been shown to be upregulated in hypoxic flies [14], [15]. Consistent with these results, we observed upregulation of all three genes after 5 hours of anoxic treatment in wild-type flies (Fig. 5a). While the expression of different genes is affected by varying concentrations [16] and exposure times of oxygen [17], many genes upregulated in hypoxia (5% O_2_) are also upregulated in anoxia (<1% O_2_) [16]. Surprisingly, we did not observe consistent elevation of these three genes in 4 day old *drd^lwf^* mutants (Fig. 5b). In *drd^lwf^* flies, *LDH* expression was significantly higher than control Canton S flies, and was elevated to the same degree as anoxic Canton S flies. However, *sima* and *tango* followed a different pattern. There was no significant difference in the expression of these genes in *drd^lwf^* flies when compared to either control or anoxic Canton S flies. Importantly, neither knockdown (Fig. 5c and d) nor rescue (Fig. 5e) of *drd* expression in the tracheae had any effect on the expression of the three genes.

**Figure 6:**
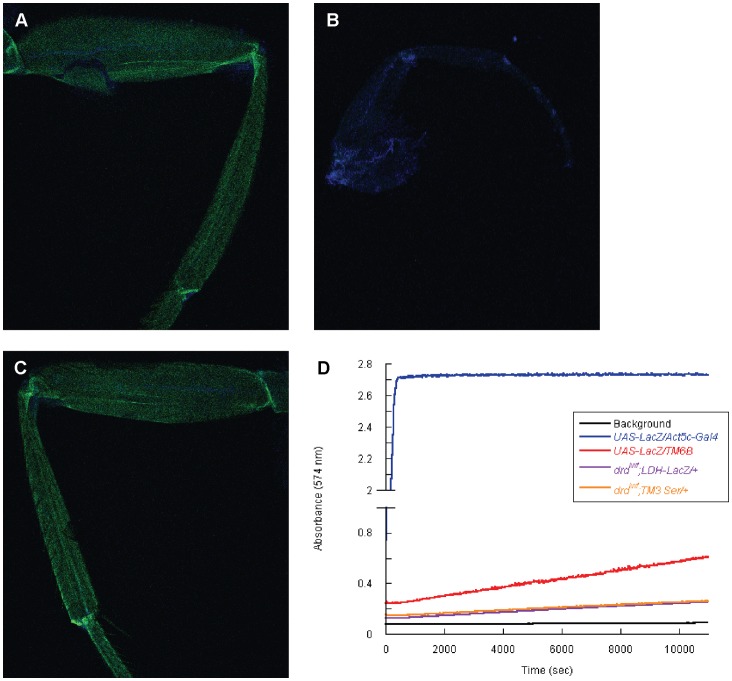
***drd^lwf^*** **flies are not hypoxic.** Induction of the *LDH-Gal4 UAS-GFP* reporter is observed in the legs of 3 day old wild-type (a) and *drd^lwf^* (c) flies after 5 hours of anoxic treatment but not in untreated *drd^lwf^* flies (b). d) β-galactosidase assays were performed on the progeny from crossing *Act5C-Gal4* males with *UAS-LacZ* females as a positive control. The *LDH-LacZ* promoter was not induced when placed on a *drd^lwf^* background. Data from a single set of homogenates are shown; identical results were seen with four additional independent sets of homogenates.

Due to the variability among the different genes observed in the qRT-PCR results, we utilized hypoxia-sensitive LDH reporter lines as an additional test of whether *drd^lwf^* flies are hypoxic. These lines contain multiple hypoxia response and cyclic AMP response elements (HRE and CRE, respectively) from the murine LDH promoter upstream of a LacZ or Gal4 (*UAS-GFP*) reporter gene and both have previously been shown to be activated by hypoxia in *Drosophila* [18]. We crossed the *LDH-Gal4 UAS-GFP* chromosome onto a *drd^lwf^* background and screened for GFP expression in 3 day old flies. GFP expression is strongly induced in the leg of anoxic wild-type flies (Fig. 6a), but there was no GFP expression observed in the leg of 3 day old *drd^lwf^* flies (Fig. 6b). To ensure that the signaling pathway leading to reporter expression is not impaired in *drd^lwf^* flies, we subjected these flies to an anoxic insult. Again, GFP expression was observed in the leg (Fig. 6c). Because of concern that low levels of GFP expression might not be visible, we also performed a more quantitative assay for using the *LDH-LacZ* reporter transgene. The reporter was placed on a *drd^lwf^* background, and β-galactosidase assays were performed on homogenates of 3 day old flies. As shown in Figure 6d, we failed to observe any β-galactosidase activity over a 3 hour time course in the *drd^lwf^;LDH-LacZ/+* flies, while positive controls exhibited robust activity.

## Discussion

Flies carrying the recessive X-chromosome mutation for *drd* exhibit early lethality, along with several seemingly unrelated phenotypes, including neurodegeneration. We set out to determine the tissue(s) necessary for the neurodegenerative phenotype and the relationship between neurodegeneration and hypoxia in *drd* mutants. We only observed neurodegeneration and early lethality when knocking down *drd* in the tracheal system. While *btl-Gal4* has been used extensively as a tracheal-specific driver, it is known to express in other tissues, most notably in embryonic midline glia [19]. However, we did not observe consistent expression of the two independent *btl-Gal4* insertions outside of the tracheae in the late pupa. In addition, knockdown of *drd* expression with the *DJ717-Gal4* driver, which is also highly expressed in the tracheae, also caused both early lethality and neurodegeneration. Taken as a whole, these data strongly suggest that *drd* expression in the tracheae is required for adult viability and integrity of the brain. Neurodegeneration was not observed in all individuals; this is perhaps not surprising given that flies were sectioned at the point of median survival such that some of the sectioned flies could have lived for many days longer.

Knockdown of *drd* in neurons, glia, or both did not cause early lethality, and thus we assume does not cause neurodegeneration. While we cannot rule out the possibility that our neuronal/glial knockdown of *drd* was incomplete and that the residual expression of *drd* in the brain was sufficient to support adult survival, we believe it more likely that *drd* expression is not required in the central nervous system for adult survival and the normal structural integrity of the brain. Consistent with this interpretation, recent immunostaining data revealed that Drd protein in the nervous system was only observed in the cone cells of the eye, while immunostaining in the tracheae was present in embryonic, larval, and pupal stages [4]. Temporally, the early lethality phenotype in adult *drd* mutants is caused by a lack of *drd* expression during pupal metamorphosis [8]. Combining our previous data with the current study, we can conclude that the neurodegenerative phenotype is caused by a lack of *drd* expression in the tracheae during the final two days of metamorphosis. It is interesting to note that in adult *drd* mutants, the tracheae are reported to lack blue fluorescence, which could be indicative of a reduction in the number of dityrosine bonds forming in the cuticle [4]. We speculate that Drd could be functioning at this developmental point in the tracheae to permit the formation of dityrosine bonds.

We have previously shown that the early adult lethality and reduced adult body mass observed in *drd* mutants are independent phenotypes [8]. In the current study, we have further separated the diverse phenotypes of *drd* mutants. Knockdown of *drd* in the tracheae results in neurodegeneration and early lethality. However, these flies have normal triglyceride and glycogen stores and a normal defecation rate, indicating that they are not starving and that food is moving normally through the gut. Moreover, rescue of *drd* expression specifically in the tracheae rescues neurodegeneration but not defecation or early lethality. We had previously hypothesized that the *drd* gut phenotype could have been a secondary consequence of neurodegeneration and the resulting loss of neuronal control over the stomodaeal valve in the cardia [7]. However, our ability to separate the gut and neurodegeneration phenotypes by tracheal-specific knockdown and rescue of *drd* disproves this hypothesis. Rather, our results indicate that neurodegeneration and gut dysfunction are independent phenotypes, with the first associated with *drd* expression in the tracheae and the tissue dependence of the second still undetermined. In addition, our data show that while the early adult lethality of *drd* mutants can be associated with neurodegeneration, neurodegeneration is not necessary for early lethality. We predict that gut dysfunction and subsequent starvation can also cause early death in *drd* mutants.

In a classic study using mosaic flies, Hotta and Benzer [20] reported that early lethality in *drd* mutants is associated with the genotype of the head, providing support for a neurodegenerative cause of death. However, the flies in this experiment were scored as mutant or wild-type based on their survival only for the first 10 days post-eclosion, and the results were interpreted based on the assumption of a single anatomical and developmental locus for *drd* expression. Given our evidence for multiple independent causes of death in *drd* mutants, we would argue that the results of Hotta and Benzer should be interpreted with caution.

As stated earlier, two hypotheses have been advanced regarding the cause of neurodegeneration in *drd* mutants: hypoxia and immature glia. While our observed link between *drd* expression in the tracheae and neurodegeneration might argue for the importance of hypoxia, we do not see consistent molecular evidence that *drd^lwf^* flies are actually hypoxic. While we observed an upregulation of *LDH* in *drd^lwf^* flies compared to Canton S flies, there was no significant difference in the expression levels of *sima* and *tango*. A previous study showed through semi-quantitative RT-PCR that six hypoxia-induced genes, including *sima* and *tango*, are upregulated in *drd^1^* flies [4]. Because we observed intermediate levels of *sima* and *tango* expression in *drd^lwf^* that was not significantly different from either control or hypoxic Canton S, there is no direct disagreement between our data and those of Kim et al. However, we showed further that tracheal knockdown or rescue of *drd* did not have any effect on the expression levels of *LDH*, *sima*, or *tango*, suggesting a lack of any association between neurodegeneration and hypoxia. Moreover, the reporter transgenes *LDH-Gal4* and *LDH-LacZ*, which are activated by the Sima/Tango transcription factor and induced by hypoxia [18] and anoxia [21], are not detectably active in our assays of *drd^lwf^* males, suggesting that these flies are not globally hypoxic. While we cannot rule out the possibility that *drd* mutant flies exhibit localized hypoxia, perhaps within specific regions of the brain, such areas of hypoxia would have to be small enough not to be detectable in our whole-fly β-galactosidase assay. Further evidence these flies are not hypoxic comes from a previous study demonstrating that even with a compromised tracheal system, *drd* mutants are still capable of satisfying the O_2_ demands required for extensive running activity [5].

If *drd* mutants are not hypoxic, why might the expression of hypoxia-induced genes be elevated? We believe that the upregulation in some of these genes is not the product of hypoxia, but rather the result of stress. The expression levels of *LDH*, *sima*, and *tango* are elevated in flies exposed to numerous stressors, including heat, heavy metals, and drugs of abuse [22]. Given the physiological insults suffered by *drd* mutants, the elevated expression of these genes is neither surprising nor a reliable indicator of hypoxia.

Our data do not address directly the proposed causal link between immature glial cells and neurodegeneration in *drd* mutants. Based on our observed connection between neurodegeneration and tracheal expression of *drd*, we would modify and extend this hypothesis. We propose that the inability of the tracheal system to develop properly is causing the stunted glial morphology observed in *drd* mutants, and that this glial defect ultimately leads to neurodegeneration. The process of glial cell maturation occurs in late pupae [3], which is the developmental period in which *drd* is required in the tracheae. Additionally, there is direct contact between all tracheae and glia in the brain [23] and the dorsal longitudinal muscle neuromuscular synapse [24]. In the neuromuscular junction, it is hypothesized that the glia function in the signaling related to gas exchange [24]. In the brain, tracheal cell maturation is dependent on glial cells [23], and the complementary relationship of glial cell maturation being dependent upon tracheal cells is possible. We propose that without proper tracheal development, glial cells fail to develop properly in *drd* mutants, ultimately resulting in neurodegeneration.

## Supporting Information

Figure S1 **Gal4 drivers that cause early lethality when** ***drd*** **is knocked down are expressed in the pupal tracheal system.** A UAS-GFP transgene present on the same chromosome as the *btl-Gal4(II)* driver was utilized to observe the driver’s expression pattern in 4 day old pupae (a). The UAS-GFP reporter was crossed with the *btl-Gal4(III)* (b) and *DJ717-Gal4* (c) drivers to visualize their expression pattern in 4 day old pupae. Images are of the ventral abdomen and white arrows point to tracheae.
(TIF)

Figure S2 **Knockdown of** ***drd*** **in the** ***DJ717-Gal4*** **pattern causes neurodegeneration.** Brain sections of 4 day old flies were stained with haematoxylin and eosin. Neurodegeneration was observed in *DJ717-Gal4/51184 UAS-Dcr-2* (b) and *DJ717-Gal4/UAS-Dcr-2 37404* (d), but not in the sibling controls (a and c, respectively). Arrows indicate holes.
(TIF)
