# Supplementary figures and images for: Neurodegeneration in Drop-Dead Mutant Drosophila melanogaster Is Associated with the Respiratory System but Not with Hypoxia
Source: PLoS One. 2013 Jul 10;8(7):e68032. doi: 10.1371/journal.pone.0068032 (PMC3707901; doi:10.1371/journal.pone.0068032)

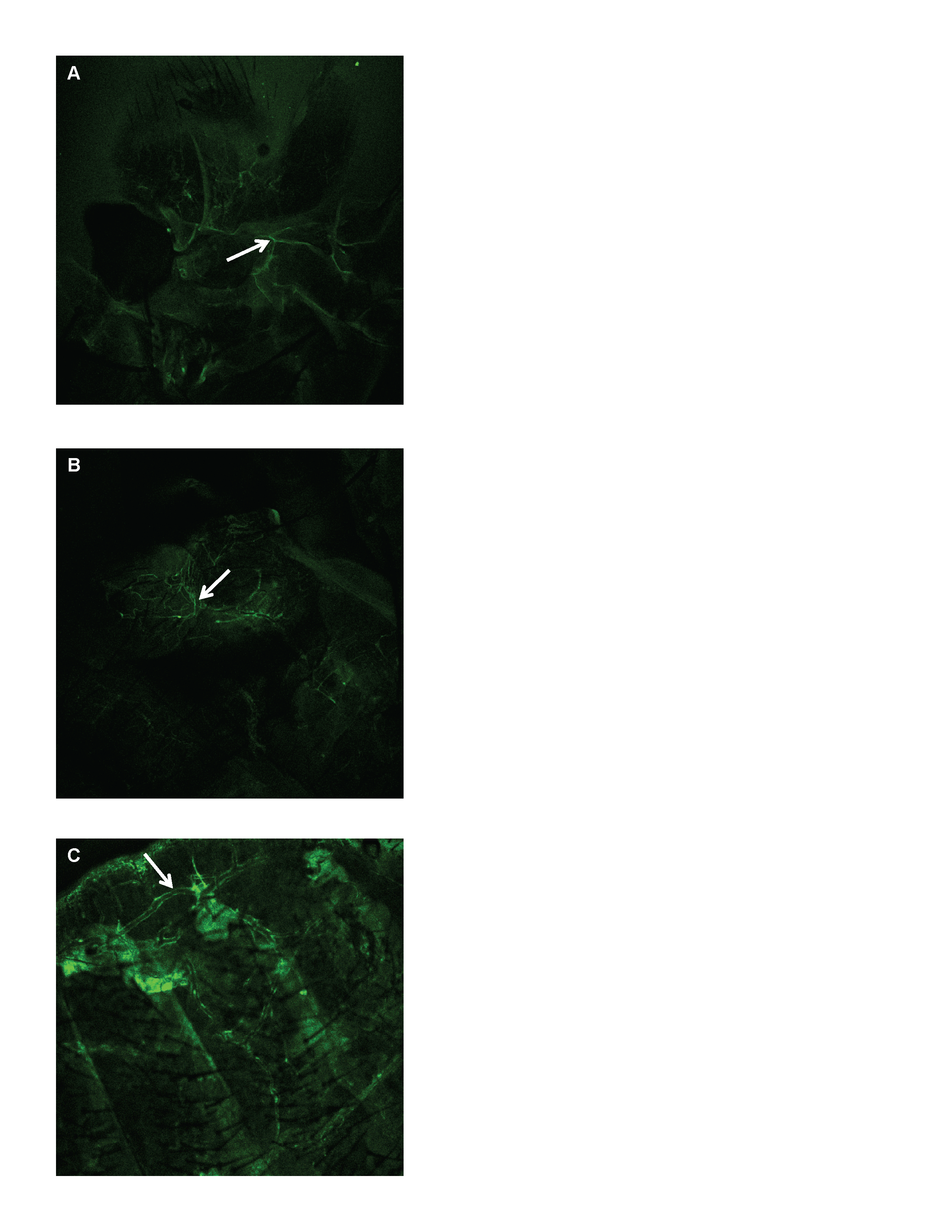

Supplement: Figure S1 — Gal4 drivers that cause early lethality when drd is knocked down are expressed in the pupal tracheal system. A UAS-GFP transgene present on the same chromosome as the btl-Gal4(II) driver was utilized to observe the driver’s expression pattern in 4 day old pupae (a). The UAS-GFP reporter was crossed with the btl-Gal4(III) (b) and DJ717-Gal4 (c) drivers to visualize their expression pattern in 4 day old pupae. Images are of the ventral abdomen and white arrows point to tracheae. (TIF) [file pone.0068032.s001.tif]

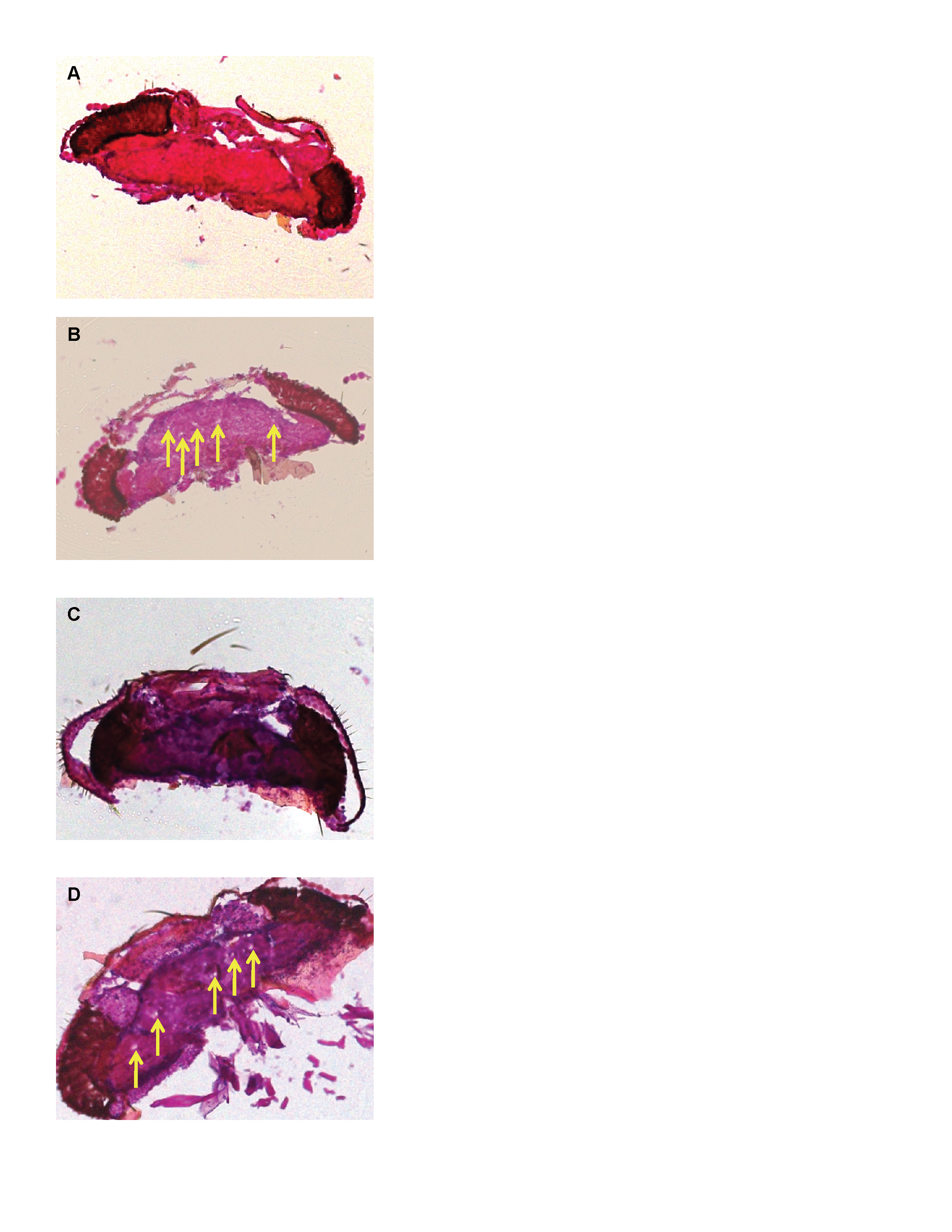

Supplement: Figure S2 — Knockdown of drd in the DJ717-Gal4 pattern causes neurodegeneration. Brain sections of 4 day old flies were stained with haematoxylin and eosin. Neurodegeneration was observed in DJ717-Gal4/51184 UAS-Dcr-2 (b) and DJ717-Gal4/UAS-Dcr-2 37404 (d), but not in the sibling controls (a and c, respectively). Arrows indicate holes. (TIF) [file pone.0068032.s002.tif]
